# Cost-effectiveness of romosozumab for the treatment of postmenopausal women at very high risk of fracture in Canada

**DOI:** 10.1007/s11657-022-01106-9

**Published:** 2022-04-26

**Authors:** Ron Goeree, Natasha Burke, Manon Jobin, Jacques P. Brown, Donna Lawrence, Björn Stollenwerk, Damon Willems, Ben Johnson

**Affiliations:** 1 Goeree Consulting Inc, Mount Hope, Canada; 2 McMaster University, Hamilton, Canada; 3 Amgen Canada Inc, 6775 Financial Drive, Suite 100, Mississauga, ON L5N 0A4 Canada; 4 Department of Medicine, Division of Rheumatology, CHU de Québec Research Centre, Laval University, Quebec City, Canada; 5 PDCI Market Access, Ottawa, Canada; 6 Amgen GmbH, Rotkreuz, Switzerland; 7 UCB Pharma, Brussels, Belgium; 8 Amgen Ltd, Uxbridge, UK

**Keywords:** Romosozumab, Osteoporosis, Cost-effectiveness, Markov model, Canada

## Abstract

***Summary*:**

This study evaluated the cost-effectiveness of 1 year of romosozumab followed by alendronate versus oral bisphosphonates alone in women with postmenopausal osteoporosis at very high risk for fracture in Canada. Results showed that romosozumab sequenced to alendronate is a cost-effective treatment option, dominating both alendronate and risedronate alone.

**Purpose:**

To demonstrate the value of romosozumab sequenced to alendronate compared to alendronate or risedronate alone, for the treatment of osteoporosis in postmenopausal women with a history of osteoporotic fracture and who are at very high risk for future fracture in Canada.

**Methods:**

A Markov model followed a hypothetical cohort of postmenopausal osteoporotic women at very high risk for future fractures, to estimate the cost-effectiveness of romosozumab and alendronate compared to oral bisphosphonates alone. A total treatment period of 5 years was assumed. Quality-adjusted life years and costs were estimated for each comparator across health states defined by different types of fragility fractures.

**Results:**

Romosozumab/alendronate was associated with a lifetime gain of 0.103 and 0.127 QALYs and a cost reduction of $343 and $3805, relative to alendronate and risedronate, respectively. These results were driven by a reduction of the number of fractures (2561 per 1000 patients, versus 2700 for alendronate and 2724 for risedronate over lifetime). Romosozumab/alendronate had the highest probability of being cost-effective, relative to alendronate and risedronate, at any willingness to pay threshold value.

**Conclusion:**

Romosozumab/alendronate was associated with reduced costs and greater benefit relative to other comparators. Probabilistic, deterministic, and scenario analyses indicate that romosozumab/alendronate represents the best value for money; the uncertainty analyses are robust, and therefore romosozumab should be considered for reimbursement by public drug plans in Canada .

## Introduction

Postmenopausal osteoporosis (PMO) is a chronic disease characterized by compromised bone strength due to bone loss that puts women at high risk of suffering debilitating fractures. Fragility fractures can result in loss of independence for patients and increased burden for themselves and their caregivers [1]. Fragility fractures are also associated with an increased risk of mortality, which may persist for several years, particularly for hip fractures [2, 3]. Patients may require long-term institutional care as a result of their fracture, with as many as 37% of patients entering long-term care in Canada following a hip fracture [4]. In Canada, the crude fracture rate in 2015 was approximately 16 fragility fractures per 1000 persons aged ≥ 50 years, and the lifetime probability of hip fracture at age 50 years was 8.9% [5]. The number of fragility fractures in those aged 50 years and older is expected to increase by 24% from 2015 to 2030 [5].

Once a postmenopausal woman has her first fracture due to osteoporosis, she is five times more likely to fracture again within a year, and her risk remains elevated over time [6]. On average, the risk of subsequent fracture is highest 1 to 2 years after the initial fracture [7–9]. In a recent real-world study in Canadian patients aged 65 years and older with a fracture, nearly 18% incurred a second fragility fracture, and the median time to second fracture was less than 2 years [10].

Osteoporotic fractures are associated with considerable direct and indirect costs. The economic burden of fragility fractures in Canada was estimated at CAD$4.6 billion in 2014; with acute care accounting for the greatest proportion (33%) of the total economic burden [4].

Recently, the Public Health Agency of Canada recognized osteoporosis as a major public health concern in Canada and highlighted the need to focus on secondary fracture prevention and its negative consequences, while facing a large care gap and a rapidly aging Canadian population [11]. The Osteoporosis Canada guidelines, last updated in 2010 (and currently being revised), focus on preventing fragility fractures and acknowledged that both antiresorptive and bone-forming agents reduce the risk of fractures in postmenopausal women at high risk of fracture [12]. Oral bisphosphonates (antiresorptive agents) are the primary first-line treatment of osteoporosis in Canada to reduce the risk of vertebral and non-vertebral fractures [13]. To facilitate absorption and avoid gastrointestinal (GI) irritation, oral bisphosphonates must be taken at least 30 min before first food, while standing and with sufficient volume of water. Low rates of persistence at 24 months have been observed with oral BPs, raloxifene, and teriparatide [14–16]. Patients not persistent on osteoporosis medications have a 40% higher risk of hip fracture compared with persistent patients [17]. Low treatment rates overall and poor persistence with BPs leave women with PMO at elevated risk of fracture.

Based on the current public reimbursement status, current treatment options for treatment-naïve post fracture patients are limited. Recent international guidelines recommend bone-forming agents as first-line therapies to reduce the fracture risk in patients at a very high risk of fracture [7, 9, 18]. However, teriparatide is not a benefit under the public drug programs outside of Quebec, and abaloparatide is not approved in Canada. Denosumab, zoledronic acid, and raloxifene are also indicated for the treatment of women with PMO; however, reimbursement is generally restricted to those patients at high risk for fracture who have failed or are intolerant to available therapies or are contraindicated to oral bisphosphonates.

Romosozumab (EVENITY®), a monoclonal antibody that binds to and inhibits sclerostin, is a bone-forming agent with a dual effect on bone, increasing bone formation and decreasing bone resorption, unlike other bone-forming agents such as teriparatide, where both bone formation and resorption are increased. This dual effect is responsible for rapid onset of action (within 1 year) on both trabecular and cortical bones, improving bone mass, structure, and strength. In treatment naïve postmenopausal women with osteoporosis and prior fragility fracture, romosozumab, sequenced to alendronate at 12 months, was found to rapidly reduce fractures within 12 months and resulted in a 48% lower risk of new vertebral fractures at 24 months compared to treatment with alendronate only [19]. Considering its clinical efficacy, further studies have evaluated the cost-effectiveness of romosozumab and sequential therapies relative to other osteoporosis therapies across multiple settings [20, 21]. Two studies conducted in Japan and Sweden concluded that romosozumab represented a cost-effective alternative treatment relative to teriparatide and alendronate as first-line treatment for postmenopausal women. These economic evaluations were conducted to provide decision-makers with high-quality evidence to determine the value for money of different treatments for osteoporosis and help inform resource allocation. However, evidence of the cost-effectiveness of romosozumab in Canada is currently unavailable.

Therefore, the objective of this study was to determine the cost-effectiveness of romosozumab and sequential therapy for the treatment of postmenopausal women at very high risk of fracture in Canada, relative to alendronate and risedronate alone, from a healthcare payer and societal perspective, using a previously validated cohort model for the treatment of PMO.

## Methods

### Target population, perspective, time horizon, and discount rate

The modelled patient population consisted of a hypothetical cohort of postmenopausal osteoporotic women who are at very high risk for future fracture. The model included patients with a mean age of 74 years (based on participants in the ARCH trial [19]). In line with the American Association of Clinical Endocrinologists/American College of Endocrinology (AACE/ACE) [7] and Endocrine Society Guidelines [8] definitions of very high risk for future fracture, the population consisted of patients with a femoral neck BMD T score ≤  − 2.5 and a history of fragility fracture. Since clinical evidence has shown that the distribution of single and multiple fractures was approximately even, the assumption was made that 50% of patients had a single previous fracture, and 50% had multiple prior fractures [22]. Considering that treatment for osteoporosis influences the risk of fractures and mortality [2], the model used a lifetime time horizon to capture all relevant benefits and costs associated with treatment [23]. All costs and health outcomes were discounted at an annual rate of 1.5% as recommended by the Canadian Agency for Drugs and Technologies in Health (CADTH) Guidelines for the Economic Evaluation of Health Technologies [23]. Alternative discount rates (e.g., 0, 3%) were considered in scenario analyses. In the reference case, the analysis was conducted from the perspective of the public healthcare payer in Canada, with a societal perspective used in a scenario analysis.

### Comparators

In the reference case analysis, the model included the following interventions:

- Romosozumab 210 mg monthly for 12 months sequenced to alendronate 70 mg weekly (romosozumab/alendronate)
- Alendronate 70 mg weekly
- Risedronate 35 mg weekly

Alendronate and risedronate were selected as comparators because they comprise the large majority of antiresorptive prescriptions in Canada [13]. Patients in the romosozumab/alendronate arm received 12 months of romosozumab (consistent with the duration specified in the product label [24]), sequenced to alendronate, which aligns with the clinical evidence from the ARCH trial [19]. Patients in all arms were assumed to be treated for a total of 5 years; a commonly recommended duration for pharmacological osteoporosis therapy [25]. The model assumed that patients were persistent with therapy in all three arms over the 5-year treatment period.

### Type of economic evaluation and model structure

A Markov cohort state transition model with a 6-month cycle length was used to assess costs and quality-adjusted life-years (QALYs) associated with romosozumab sequenced to alendronate (romosozumab/alendronate) compared with alendronate alone or risedronate alone. The model structure is based on the model developed by the International Osteoporosis Foundation, which has been widely used as a basis for economic analyses of osteoporosis [26–32]. Markov models are considered appropriate methodologies for this therapeutic area, considering that osteoporosis is a chronic condition and involves a continuous risk over time [28–33].

**Fig. 1:**
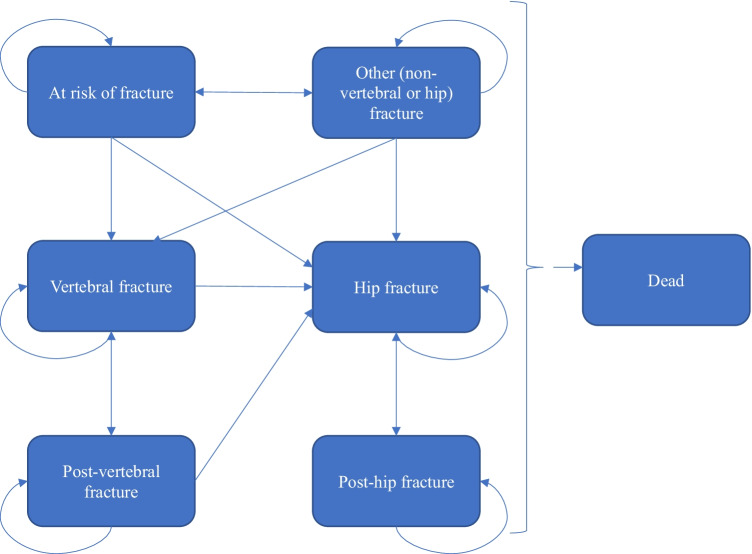
Structure of the Markov cohort model. Arrows to the health state “dead” excluded for simplification

Seven Markov health states were considered (Fig. 1): at risk of fracture (i.e., baseline), clinical vertebral fracture, post clinical vertebral fracture, hip fracture, post-hip fracture, “other” fragility fracture (i.e., non-vertebral non-hip fragility fracture), and death. All patients were assumed to be at risk of fracture at baseline. During each cycle, patients had a probability of sustaining a fracture, remaining in the baseline state, or dying. Patients who sustained a fracture transitioned to any of the three health states depending on the fracture type (hip, vertebral, or other fracture). After 1 year in the “other” fracture state, patients who did not sustain another fracture returned to the baseline at-risk health state. After 1 year in the hip and vertebral fracture states, patients who did not sustain another fracture transitioned to the “post-hip fracture” and “post-vertebral fracture” states, respectively.

The model was built with a hierarchical structure based on the severity of fracture types, with hip being the most severe, followed by vertebral and “other.” The structure assumed that patients were only allowed to transition to more severe health states. For example, patients who sustained a hip fracture could not subsequently transition to vertebral or “other” fracture states. This assumption allowed capturing long-term costs and HRQoL with the post-hip or post-vertebral fracture states [29, 31]. However, the hierarchical structure does not explicitly count subsequent fractures further down in the hierarchy, and therefore the model may result in an underestimation of fracture incidence. To correct for this, as in previous adaptations, lower hierarchy fractures were estimated separately by multiplying the number of subjects in each higher hierarchy state with the incidence rate of the lower hierarchy fracture type in the model population [20, 27].

### Efficacy and clinical inputs

(i) BMD and fracture risk (general population and PMO high risk)

The risk of sustaining a fracture in the model depended on (i) the risk for an individual in the general population of incurring a fracture, (ii) the increased fracture risk associated with osteoporosis, and (iii) a risk reduction, if any, attributed to treatment. The general population risk depended on age and gender. The risk of fracture relative to the general population depended on age, bone mineral density (BMD), and prior fracture prevalence. Age-specific general population fracture rates were taken from Canadian sources (as shown in Appendix Table 5). These values were linearly interpolated or extrapolated as required to produce fracture rates for each year of age (Appendix Table 5). To estimate fracture risks in untreated patients with PMO at very high risk for future fracture, general population fracture rates were adjusted for the lower BMD T scores and higher prevalence of previous fracture in the modelled population. Fracture risks for the target population were adjusted with relative risks of subsequent fracture in patients with a prior vertebral fracture and relative risks of subsequent fracture per standard deviation decline in BMD, as described in previous economic evaluations [20, 30].

(ii) Efficacy

For patients receiving treatment, treatment efficacy data (relative risks of fracture) were applied to fracture rates in untreated very high-risk postmenopausal osteoporotic patients. Efficacy data used in the model provided relative risks (RRs) of hip, new vertebral, and nonvertebral fracture separately. RRs of new vertebral fracture were used to inform treatment-specific efficacy in preventing vertebral fractures, while RRs of nonvertebral fracture were used to inform efficacy for “other” fractures (comprising wrist, and all other non-hip, non-vertebral fractures). Hip fracture efficacy was directly informed by treatment-specific RRs of hip fracture.

For the alendronate and risedronate arms, RRs of hip, vertebral, and nonvertebral fracture versus placebo were applied to fracture rates in untreated patients for the duration of treatment (5 years). These data were obtained from a network meta-analysis of randomized controlled trials (Table 1) [34]. For the romosozumab/alendronate arm, RRs of fracture versus placebo were established indirectly from two sources: a comparison of romosozumab with alendronate from the ARCH trial [19, 35] and the comparison of alendronate with placebo [34]. Because of their different modes of action, patterns of treatment benefit over time likely differ for regimens containing a bone-forming agent and regimens consisting of an antiresorptive agent alone. Therefore, RRs of fracture for romosozumab/alendronate versus alendronate were calculated time-dependently. To do this, parametric survival curves were fit to time-to-event data for hip and nonvertebral fractures from the ARCH trial. For each regimen and fracture type, fracture incidence in each 6-month period was calculated from the selected parametric survival functions. The survival functions were selected based on best fit, defined by the Akaike information criterion (AIC). A scenario analysis was considered to select survival functions based on the Bayesian information criterion (BIC) (Appendix Table 6). These values were used to calculate RRs of hip and nonvertebral fracture for romosozumab/alendronate versus alendronate in each 6-month model cycle over the 5-year treatment period (Table 1). Survival models were fitted separately by arm to allow changing fracture incidence over time.

**Table 1:** Relative risks for hip, vertebral, and non-vertebral fractures per cycle (romosozumab/alendronate relative to alendronate alone) [19, 34, 35]

Model cycle^a^	Hip fracture	New vertebral fracture	Non-vertebral fracture
1	0.89	0.64	0.70
2	0.60	0.64	0.75
3	0.56	0.38	0.79
4	0.56	0.38	0.85
5	0.57	0.38	0.90
6	0.58	0.38	0.96
7	0.59	0.38	1.02
8	0.60	0.38	1.08
9	0.62	0.38	1.15
10	0.63	0.38	1.23
**Comparison**	**Hip fracture** **(95% CIs)**	**New vertebral fracture (95% CIs)**	**Non-vertebral fracture (95% CIs)**
Alendronate vs placebo	0.61 (0.42 to 0.90)	0.57 (0.45 to 0.71)	0.84 (0.74 to 0.94)
Risedronate vs placebo	0.73 (0.58 to 0.92)	0.61 (0.48 to 0.78)	0.78 (0.68 to 0.89)

^a^Cycle-length 6 months

*CI*, confidence interval; *vs*, versus

Note: uncertainty around estimates is not shown, since RRs of hip fracture and non-vertebral fracture are derived from parametric survival curves (estimated from Barrionuevo et al. [34]), the intercept and scale of which were sampled in probabilistic sensitivity analysis to account for uncertainty

(iii) Treatment offset time

The fracture reduction benefit of pharmacological osteoporosis treatment does not disappear immediately following discontinuation, but rather persists for some time (i.e., the “offset time”). A clinical study in which patients received 5 years’ alendronate treatment followed by 5 years’ placebo found that mean BMD remained at or above pre-treatment levels [36], suggesting that treatment benefit persists for a substantial period. Therefore, the assumption was made that the fracture reduction benefit of treatment declines linearly to 0 over the length of time for which a patient was treated. That is, the treatment offset period lasts for 5 years.

(iv) Mortality

General population all-cause mortality was informed by life tables for females in Canada from Statistics Canada [37]. The model also accounted for the increased risk of mortality following a fracture. Two key assumptions were made regarding mortality following osteoporosis-related fractures: (i) 30% of the excess mortality following a fracture was attributable to the fracture itself, in line with previous analyses [29–31] and (ii) the increased risk of mortality after hip and vertebral fractures was assumed to last for 8 years as per previous analyses [29–31]. This duration of excess mortality only applied to hip and vertebral fractures as other fractures were assumed to only have effects in the first year of fracture. Age-specific RRs of mortality in the first year after hip, vertebral, and other fracture, and in the second and following years after hip and vertebral fracture for Canadian women were sourced from Morin et al. [2]. (Appendix Table 7).

### Health-related quality of life

To account for the HRQoL loss due to fracture, in the first year after hip, vertebral, and other fracture, and for the second and subsequent years after hip and vertebral fracture, utility multipliers were applied to utilities of the general population. Data specific to subsequent “other” fractures were not available. Therefore, the utility multiplier in the first year after “other” fracture was assumed to correspond to that of a distal forearm fracture. These values were taken from Svedbom et al. [38], an analysis of HRQoL from the International Costs and Utilities Related to Osteoporotic Fractures Study (ICUROS), which recorded HRQoL before and after fracture, for different fracture locations. In a scenario analysis, Canadian-specific HRQoL inputs were derived from HRQoL at different time points to estimate disutilities associated with fractures [39]. Health-related quality of life and utility parameters are summarized in Table 2.

**Table 2:** Health-related quality of life, utilities, and drug, treatment monitoring/administration, direct medical, and long-term costs

Input		Value	Source
**Female general population health-related quality of life by age**			
**Age (years)**			Guertin et al. 2018 [40]
50–54		0.842	
55–59		0.830	
60–64		0.841	
65–69		0.837	
70–74		0.831	
75–79		0.778	
80–84		0.736	
85 +		0.616	
**Health-related quality of life multipliers by fracture type**			
**Fracture type**			Svedbom 2018 [38]
Hip fracture, 1st year		0.55	
Hip fracture, 2nd and subsequent years		0.86	
Vertebral fracture, 1st year		0.68	
Vertebral fracture, 2nd and subsequent years		0.85	
Other fracture, 1st year		0.83	
**Annual drug costs**			
**Romosozumab**	Unit cost	$328.39	Manufacturer
	Dosing frequency	Monthly (2 syringes per dose)	
	Annual cost	$7881	
**Alendronate**	Unit cost	$2.10	Ontario Drug Benefit Formulary [41]
	Dosing frequency	Weekly	
	Annual cost	$109	
**Risedronate**	Unit cost	$1.98	
	Dosing frequency	Weekly	
	Annual cost	$103	
**Annual treatment monitoring/administration costs**			
**Romosozumab**	BMD measurements	$42	Ontario Health Insurance Plan Schedule of Benefits and Fees [42]
	Physician visits	$77	
	Nurse visits	$191	
	Annual cost	$310	
**Alendronate**	BMD measurements	$42	
	Physician visits	$77	
	Nurse visits	$0	
	Annual cost	$119	
**Risedronate**	BMD measurements	$42	
	Physician visits	$77	
	Nurse visits	$0	
	Annual cost	$119	
**Direct medical costs of fracture**			
**Hip fracture**	First year after fracture—50–59 years	$21,898	Metge et al. (2010) [43]
	First year after fracture—60–69 years	$20,875	
	First year after fracture—70–79 years	$27,512	
	First year after fracture—80–89 years	$29,782	
	First year after fracture—90 + years	$27,398	
	Second and following years after fracture	$5171	Goeree et al. (2006) [44]
**Vertebral fracture**	First year after fracture—50–59 years	$11,427	Metge et al. (2010) [43]
	First year after fracture—60–69 years	$15,342	
	First year after fracture—70–79 years	$18,600	
	First year after fracture—80–89 years	$23,683	
	First year after fracture—90 + years	$28,341	
	Second and following years after fracture	$235	Goeree et al. (2006) [44]
**Other fracture**	First year after fracture—50–59 years	$2025	Metge et al. (2010) [43]
	First year after fracture—60–69 years	$2709	
	First year after fracture—70–79 years	$7268	
	First year after fracture—80–89 years	$15,175	
	First year after fracture—90 + years	$20,203	
**Long-term care costs**			
Proportion of patients entering long-term care after hip fracture		37%	Hopkins et al. 2016 [4]
Daily cost of long-term care		$184.96	AdvantAge Ontario [45]

All costs are expressed in 2020 CAD

*BMD*, bone mineral density

### Resource use and costs

The model included drug acquisition costs, treatment monitoring/administration costs, direct medical costs due to fracture, and long-term care costs (Table 2). Additionally, broader societal costs (including lost productivity costs and patient out-of-pocket costs) were included in a scenario analysis. Lost productivity was estimated based on the mean hourly wage of females 55 years or older, working full and part time in Canada. The value of lost productivity associated with fractures was estimated based on the average time off from work due to each fracture type (Appendix Table 8). For out-of-pocket costs, an assumption was made that BMD measurements, physician visits, and nurse visits were associated with a $20 parking and travel fee. All costs were expressed in Canadian dollars (CAD), inflated to 2020 values where required using the Consumer Price Index (CPI) for Canada [46]. Drug acquisition costs were obtained from the manufacturer for romosozumab and from the list prices on the Ontario Drug Benefit Formulary [41] for antiresorptive agents, using the lowest available unit price for the weekly oral dose of 70 mg and 35 mg for alendronate and risedronate respectively. A conservative assumption was made that patients were fully persistent with therapy in all three arms over the 5-year treatment period, due to the current lack of real-world persistence data for romosozumab sequenced to an antiresorptive and the inherent limitations of discontinuation data from RCTs. In reality, it is known that persistence with osteoporosis treatments is imperfect [47]. Discontinuation data from the randomized controlled ARCH trial are unlikely to represent rates in practice, and therefore full persistence with all treatments was assumed in the model. Wholesaler upcharge and pharmacist dispensing fees were not considered.

For treatment monitoring/administration costs, the model assumed that patients receiving treatment incurred the cost of a physician visit once a year, and the cost of a BMD measurement every 2 years, as per Ontario Health Insurance Plan Schedule of Benefits and Fees [42]. The model assumed that 85% of patients treated with romosozumab required a monthly nurse visit for subcutaneous injection administration. Based on feedback from Canadian clinicians, it was conservatively assumed that the remaining 15% of patients would self-administer romosozumab requiring 2 nurse visits in total (one training visit and one follow-up visit). The cost per nurse visit was estimated assuming a 20-min appointment and based on an hourly wage of $46.31 as per the 2020 Ontario Nurses’ Association Collective Agreement [48]. Age-specific costs in the first year after hip, vertebral, and other fracture in Canada were informed by Metge et al. [43]. Values used in the model comprised total incremental healthcare costs for the year following fracture, versus the year pre-fracture. The cost of “other” fracture was calculated as a weighted average of wrist and humerus fracture.

### Analysis

The model estimated total discounted lifetime costs and QALYs for each intervention, with cost-effectiveness assessed by dominance and in terms of incremental cost-utility ratios (ICURs). Reference case results were assessed through a probabilistic model with 5000 stochastic iterations, where parameters were varied simultaneously according to distributions representing their uncertainty [49]. Cost-effectiveness acceptability curves (CEACs) were derived to summarize the proportion of probabilistic iterations in which each comparator was cost-effective across a range of willingness to pay per QALY-gained thresholds. In addition, sensitivity analyses using the deterministic model were performed to assess the sensitivity of results to changes in individual parameters. Parameters were varied using published confidence intervals or standard errors, where available, and by 25% above and below point estimates where measures of uncertainty were unavailable. Cost-effectiveness was assessed with the incremental net monetary benefit (INMB) for each deterministic sensitivity analysis.

Scenario analyses were conducted to assess the impact of using alternative model assumptions. For each scenario analysis, 5000 iterations of the probabilistic model were conducted. The first scenario tested model structural uncertainty by assuming an alternative treatment sequence, where romosozumab sequenced to risedronate was compared with alendronate alone and risedronate alone. The efficacy of romosozumab/risedronate was assumed to be equivalent to that of romosozumab/alendronate in the first two cycles of the model (i.e., for the duration of romosozumab treatment). For cycles 3 to 10, cycle-specific efficacy of romosozumab/risedronate versus placebo was estimated by applying RRs of fracture for romosozumab/alendronate versus alendronate to RRs for risedronate versus placebo [34]. Additional scenario analyses around modelling assumptions were considered to account for a societal perspective and indirect costs, annual discount rates of 0 or 3%, treatment efficacy rates estimated from parametric models, modified treatment offset time (1 year), increased excess mortality duration, Canadian fracture disutilities, alternative cost for risedronate (Actonel DR), and reduced duration of fracture reduction benefits associated with romosozumab/alendronate. Cost-effectiveness was estimated across the three comparators for each scenario.

## Results

### Reference case results

The total and disaggregated reference case model results are presented in Table 3. Romosozumab/alendronate yielded the most discounted QALYs and lowest total cost (8.454 and $86,314, respectively), and was associated with a lifetime gain of 0.103 and 0.127 QALYs and a cost reduction of $343 and $3805, relative to alendronate and risedronate, respectively. Although drug costs were highest for romosozumab/alendronate ($8259 vs. $521 for alendronate and $491 for risedronate), the total cost incurred by patients treated with romosozumab/alendronate was lower relative to alendronate and risedronate ($86,314, $86,656, $90,119, respectively). Therefore, the improvements in QALYs and cost reductions for romosozumab/alendronate were driven by a reduction of the expected number of fractures (2561 per 1000 patients versus 2700 for alendronate and 2724 for risedronate). Consequently, alendronate and risedronate were dominated by romosozumab/alendronate. Despite similar annual drug costs of alendronate and risedronate, the total discounted incremental costs of romosozumab/alendronate compared to risedronate (− $3805) was lower than the comparison with alendronate (− $343) due to the differences in hip fracture incidence rates.

**Table 3:** Reference case disaggregated and fully incremental cost-effectiveness results

Outcome	Romosozumab/alendronate	Alendronate	Risedronate	Incremental (romosozumab/alendronate vs. alendronate)	Incremental (romosozumab/alendronate vs. risedronate)
**Lifetime fracture incidence per 1000 patients**					
Hip fracture	550	588	607	− 38	− 57
Vertebral fracture	715	795	810	− 80	− 96
Other fracture	1296	1317	1306	− 21	− 11
**Total**	2561	2700	2724	− 140	− 163
**Costs**					
**Fracture costs**					
Hip fracture	$69,646	$77,975	$82,016	− $8330	− $12,371
Vertebral fracture	$13,366	$14,775	$14,954	− $1409	− $1588
Other fracture	$13,710	$13,424	$13,249	$286	$461
**Total fracture costs**	$96,721	$106,175	$110,219	− $9453	− $13,497
**Drug cost**	$8259	$521	$491	$7737	$7768
**Treatment monitoring/administration cost**	$757	$566	$566	$191	$191
**Total cost (undiscounted)**	$105,737	$107,262	$111,276	− $1525	− $5539
**Total cost (discounted)**	$86,314	$86,656	$90,119	− $343	− $3805
**QALYs and life years**					
Life years (undiscounted)	14.442	14.422	14.418	0.020	0.024
Life years (discounted)	12.650	12.633	12.629	0.017	0.021
QALYs (undiscounted)	9.510	9.394	9.366	0.117	0.144
QALYs (discounted)	8.454	8.351	8.327	0.103	0.127
**Cost-effectiveness**					
ICUR	-	-	-	Dominant	Dominant

*QALY*, quality-adjusted life year; *ICUR*, incremental cost-utility ratio

**Fig. 2:**
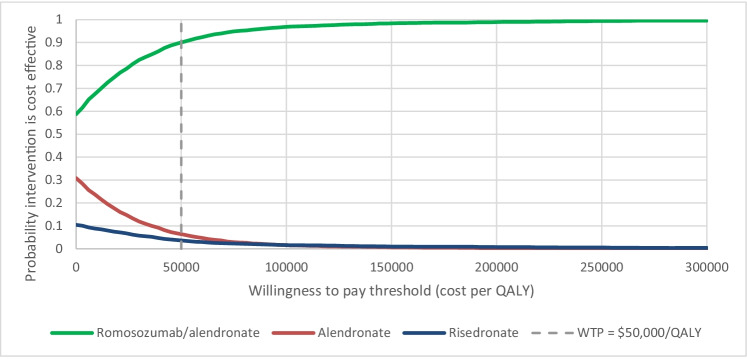
Cost-effectiveness acceptability curves. *QALY*, quality-adjusted life year; *WTP*, willingness to pay. CEACS show the number of probabilistic iterations in which each intervention is cost-effective over a range of cost-effectiveness thresholds

Results are also shown for all three comparators as cost-effectiveness acceptability curves (CEACs) (Fig. 2) and a scatter plot of the 5000 iterations (Appendix Figure 3). The number of iterations in which each intervention was found to be cost-effective is displayed over a range of cost per QALY-gained thresholds. These results show that, at a willingness to pay threshold of $50,000 per QALY for example, romosozumab/alendronate was the optimal intervention (in terms of net monetary benefit) in 92.0% of probabilistic iterations. Additionally, romosozumab/alendronate had the highest probability of being cost-effective, relative to alendronate and risedronate, at any willingness to pay threshold value.

### Sensitivity analysis

Deterministic sensitivity analysis results demonstrated that the model outcomes were most sensitive to fracture reduction efficacy and parameters relating to the cost of long-term care and proportion of patients entering long-term care after hip fracture. However, romosozumab/alendronate was consistently cost-effective, as it represented the highest NMB relative to alendronate and risedronate for each run of the one-way sensitivity analysis (see Tornado diagrams in Appendix Figures 4 and 5).

### Scenario analyses

The incremental costs, QALYs, and ICUR of romosozumab/alendronate relative to alendronate and risedronate for all scenarios are presented in Table 4. Most scenarios yielded similar results to the reference case, where romosozumab/alendronate yielded additional QALYs and fewer costs relative to alendronate and risedronate (i.e., remained the dominant intervention). However, the first scenario analysis that considered a romosozumab/risedronate sequence, represented additional costs ($1322) relative to alendronate alone (ICUR of $14,209 per QALY gained). Therefore, although not dominant, romosozumab/risedronate would be cost-effective relative to alendronate at a willingness to pay threshold of $50,000 per additional QALY gained. Finally, alendronate also had fewer costs compared with romosozumab/alendronate when treatment offset time was defined at 1 year (ICUR = $21,321). Risedronate was consistently dominated throughout all scenario analyses.

**Table 4:** Scenario analysis results—romosozumab/alendronate versus alendronate and risedronate (discounted results)

	∆ Costs	∆ QALYs	ICUR
**Scenario (romosozumab/alendronate vs. alendronate)**			
**Reference case**	− $343	0.103	Dominant
Romosozumab sequenced to risedronate	$1322	0.093	$14,209
Societal perspective	− $322	0.103	Dominant
Discount rate of 0% per annum for costs and health outcomes	− $1373	0.116	Dominant
Discount rate of 3% per annum for costs and health outcomes	$609	0.091	$6707
Parametric models with the lowest BICs used to specify fracture incidence in time-dependent efficacy calculations	− $1002	0.113	Dominant
Parametric models with the second-lowest AICs used to specify fracture incidence in time-dependent efficacy calculations	− $1106	0.112	Dominant
Treatment offset time of 1 year	$1751	0.082	$21,321
Duration of excess mortality following hip and vertebral fracture set to 5 years	− $333	0.102	Dominant
Duration of excess mortality following hip and vertebral fracture set to 10 years	− $425	0.103	Dominant
Proportion of excess mortality due to fracture event set to 10%	− $650	0.097	Dominant
Proportion of excess mortality due to fracture event set to 50%	− $188	0.109	Dominant
Only excess mortality in the first year after hip fracture considered	− $488	0.097	Dominant
Disutilities taken from Tarride 2016	− $354	0.131	Dominant
**Scenario (romosozumab/alendronate vs. risedronate)**			
**Reference case**	− $3805	0.127	Dominant
Romosozumab sequenced to risedronate	− $2091	0.117	Dominant
Societal perspective	− $3806	0.127	Dominant
Discount rate of 0% per annum for costs and health outcomes	− $5458	0.144	Dominant
Discount rate of 3% per annum for costs and health outcomes	− $2423	0.113	Dominant
Parametric models with the lowest BICs used to specify fracture incidence in time-dependent efficacy calculations	− $4448	0.137	Dominant
Parametric models with the second-lowest AICs used to specify fracture incidence in time-dependent efficacy calculations	− $4468	0.136	Dominant
Treatment offset time of 1 year	− $754	0.101	Dominant
Duration of excess mortality following hip and vertebral fracture set to 5 years	− $3659	0.126	Dominant
Duration of excess mortality following hip and vertebral fracture set to 10 years	− $3877	0.127	Dominant
Proportion of excess mortality due to fracture event set to 10%	− $4060	0.119	Dominant
Proportion of excess mortality due to fracture event set to 50%	− $3484	0.134	Dominant
Only excess mortality in the first year after hip fracture considered	− $3881	0.120	Dominant
Drug cost of ACTONEL DR ($617.00 per year) used for risedronate	− $6333	0.128	Dominant
Disutilities taken from Tarride 2016	− $3841	0.164	Dominant

*AIC*, Akaike information criterion; *ARCH*, Active ContRolled FraCture Study in Postmenopausal Women with Osteoporosis at High Risk of Fracture (phase III study); *ICUR*, incremental cost-utility ratio; *QALY*, quality-adjusted life-year; *BIC*, Bayesian information criterion

## Discussion

### Summary of findings

This economic evaluation assessed the lifetime cost-effectiveness of 1 year of romosozumab sequenced to 4 years of alendronate versus alendronate alone (5 years) and risedronate alone (5 years), for the treatment of osteoporosis in postmenopausal women in Canada with a history of osteoporotic fracture and who are at very high risk for future fracture. The Markov model employed clinical and economic inputs to estimate the incidence of several fracture types and their associated effects under specific lines of treatment. Treatment with romosozumab/alendronate was associated with the most QALYs and lowest costs, relative to the comparators. Despite having a higher drug and treatment management cost, romosozumab/alendronate produced an overall cost reduction versus both comparators from a healthcare payer perspective, due to a reduction in overall fractures and fracture-related costs. Furthermore, deterministic, probabilistic, and scenario analyses evaluated the robustness of the conclusions after accounting for structural and parameter uncertainty. In general, the parameters which had the largest impact on cost-effectiveness results were those relating to treatment efficacy as these inputs drive differences in fracture incidence between arms. Romosozumab/alendronate had the highest probability of being cost-effective at a willingness to pay threshold of $50,000 per additional QALY gained. Whereas, the current first-line treatments, alendronate or risedronate, were the optimal intervention (in terms of net monetary benefit) in less than 10% of probabilistic iterations.

### Contribution to the literature

Although the cost-effectiveness of romosozumab has been previously estimated in different countries, this evaluation contributes novel evidence to the pharmacoeconomic literature of osteoporosis in Canada. A study conducted by Hagino et al. employed a similar model to demonstrate the cost-effectiveness of romosozumab in the Japanese context [20]. However, romosozumab was compared with teriparatide, both sequenced to alendronate, in women with severe PMO previously treated with bisphosphonates. Additionally, it employed BMD efficacy data from the STRUCTURE trial rather than fracture outcomes to inform relative efficacy. Another study, conducted by Sӧreskog et al., assessed the cost-effectiveness of romosozumab/alendronate compared with alendronate alone from a Swedish societal perspective [21]. Romosozumab followed by an antiresorptive was cost-effective compared to an antiresorptive alone despite the substantial price difference; however, the study was primarily designed to present a novel cost-effectiveness model framework that incorporated recency of fracture and treatment sequencing. Whereas, our evaluation provides evidence of the cost-effectiveness of romosozumab relative to an additional comparator (i.e., risedronate), and considering an alternative treatment sequence (romosozumab/risedronate). The results of these economic evaluations suggest that romosozumab has a relatively high probability of being cost-effective relative to bone-forming agents and antiresorptive agents, in three different countries. Furthermore, we acknowledge that different model types, such as patient-level simulation models, have been used to assess the cost-effectiveness of bone-builders [21]. However, previous cost-effectiveness results were consistent with the cohort modelling approach which we applied.

### Strengths and limitations

As with all analyses based on economic models, this evaluation has a number of limitations. First, the hierarchical nature of the model can lead to an underestimation of the number of vertebral and other fractures. However, an adjustment function was introduced to correct for the omitted lower hierarchy fractures. Second, there is uncertainty in the duration of treatment “offset time”—the duration of fracture reduction benefit after treatment discontinuation. While the duration of the offset time was based on clinical evidence [36], it is not possible to precisely quantify the duration of the offset time. This assumption was tested through scenario analyses. Third, there is uncertainty in the duration of excess mortality following fracture. This uncertainty was also tested in scenario analyses, with results showing that romosozumab/alendronate remained cost-effective in all cases. Fourth, international data were used where appropriate local data were not available. These included HRQoL loss due to fracture, the baseline age of the population, and RRs used to adjust general population fracture rates for fracture history and BMD. Fifth, the efficacy of romosozumab sequenced to risedronate (included as a scenario analysis) assumed that the additional fracture reduction benefit of romosozumab/risedronate versus risedronate was equivalent to that of romosozumab/alendronate versus alendronate. However, this cannot be confirmed in the absence of direct RCT evidence. In addition, the lack of real-world persistence data for romosozumab and comparators is an inherent limitation of the analysis. Finally, the model extrapolated the efficacy data for romosozumab/alendronate versus alendronate observed in the ARCH trial over the 5-year treatment period.

### Policy implications and future work

Despite the limitations, this analysis provides clear evidence of the cost-effectiveness of romosozumab sequenced to alendronate versus alendronate and risedronate alone, with results that are robust to alternative deterministic and scenario analyses. There are several areas where further research is required to facilitate future cost-effectiveness analyses of romosozumab and osteoporosis treatments in general. Research is required to quantify the fracture reduction efficacy of romosozumab sequenced to an antiresorptive agent beyond the duration observed in the ARCH trial. Furthermore, osteoporosis models usually lack empirical data on the duration of fracture reduction benefit after treatment cessation. In this analysis, although romosozumab/alendronate remained cost-effective when the treatment offset time was reduced to 1 year, the number of fractures avoided versus antiresorptive agents alone was reduced, resulting in a positive ICUR of $9408 versus alendronate. Considering that results are sensitive to treatment offset time, further research in this area would help reduce this uncertainty. Third, research into the real-world persistence associated with romosozumab and subsequent antiresorptive treatment is needed. Given its longer dosing interval, it is likely that the persistence of romosozumab would be superior to that of oral alendronate and risedronate [47]. However, quantification of this persistence is required to inform economic analyses. Additionally, local data sources are required to inform the model, specifically around HRQoL. Canadian-specific HRQoL following fracture was estimated by Tarride et al. [39]; however, the population was restricted to patients in long-term care or receiving home care. Although these data were not used to inform the base case, they were tested in a scenario analysis. As such, further local evidence would improve the generalizability of future economic analyses of osteoporosis therapies in Canada and settings with close similarities in their health system such as the UK, Australia, and Western Europe. Lastly, further research on the effectiveness of treatment in other populations (i.e., male osteoporosis, patients with secondary forms of osteoporosis) could inform the potential to extrapolate the results of this analysis.

## Conclusion

This is the first economic model that evaluates the cost-effectiveness of romosozumab/alendronate for the treatment of postmenopausal osteoporosis in Canada. Compared with alendronate and risedronate, romosozumab/alendronate consistently yielded cost savings and higher health benefits in postmenopausal women with a history of osteoporotic fracture and who are at very high risk for future fracture. This was due to favorable fracture efficacy, which led to a cost reduction from avoided fractures and a QALY gain compared with antiresorptive agents alone. Probabilistic, deterministic, and scenario analyses indicate that romosozumab/alendronate is likely to be cost-effective at any decision-maker threshold, including the commonly quoted $50,000 per QALY gained in Canada. Romosozumab/alendronate was associated with reduced costs and greater benefit, dominating other comparators. Given these results, romosozumab/alendronate should be considered for reimbursement by public drug plans in Canada for the treatment of osteoporosis in postmenopausal women with a history of osteoporotic fracture and who are at very high risk for future fracture.

## Figures and Tables

**Fig. 3:**
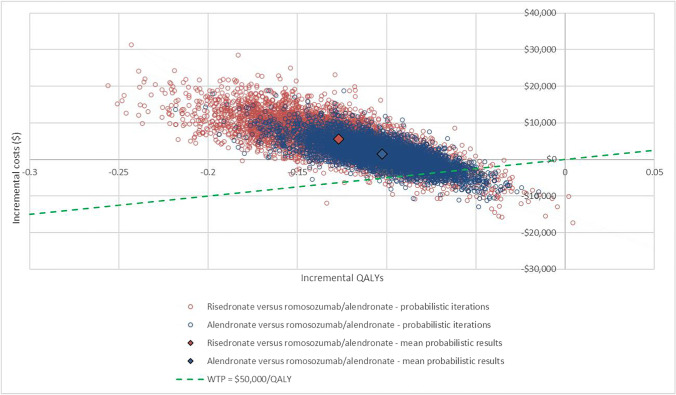
Scatter plot of probabilistic results (comparators versus romosozumab/alendronate (discounted). *QALY*, quality-adjusted life year; *WTP*, willingness to pay

**Fig. 4:**
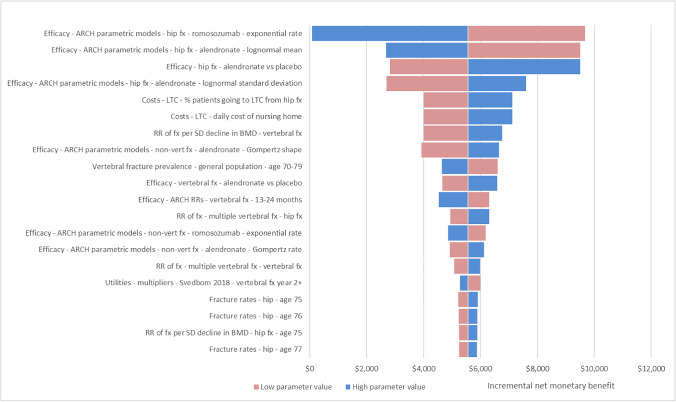
Tornado diagram of deterministic sensitivity analyses. Romosozumab/alendronate versus alendronate—discounted incremental net monetary benefit at a threshold of $50,000/QALY. *ARCH*, Active ContRolled FraCture Study in Postmenopausal Women with Osteoporosis at High Risk of Fracture (phase III study); *BMD*, bone mineral density; *Fx*, fracture; *LTC*, long-term care; *QALY*, quality-adjusted life year; *RR*, relative risk; *SD*, standard deviation; *vert*, vertebral

**Fig. 5:**
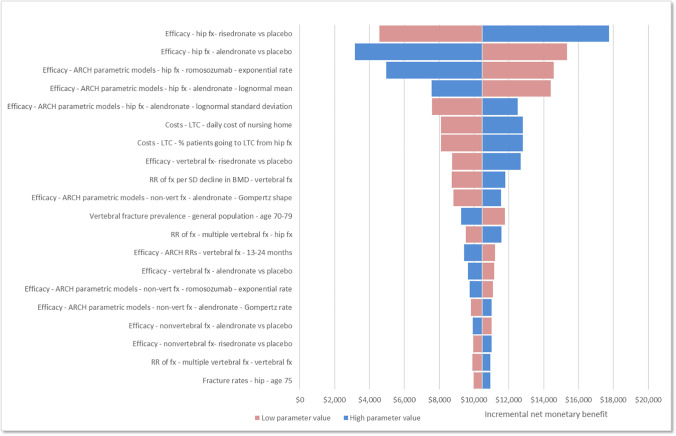
Tornado diagram of deterministic sensitivity analyses. Romosozumab/alendronate versus risedronate—discounted incremental net monetary benefit at a threshold of $50,000/QALY. *ARCH*, Active ContRolled FraCture Study in Postmenopausal Women with Osteoporosis at High Risk of Fracture (Phase III Study); *BMD*, bone mineral density; *Fx*, fracture; *LTC*, long-term care; *QAL*Y, quality-adjusted life year; *RR*, relative risk; *SD*, standard deviation; *vert*, vertebral

## Data Availability

Not applicable.

## Appendix

**Table 5:** General population annual fracture rates

Age (years)	Hip fracture	Vertebral fracture	Other fracture
50	0.0001	0.0006	0.0053
51	0.0002	0.0007	0.0057
52	0.0002	0.0007	0.0062
53	0.0002	0.0008	0.0067
54	0.0003	0.0008	0.0072
55	0.0003	0.0009	0.0077
56	0.0004	0.0010	0.0082
57	0.0004	0.0011	0.0087
58	0.0005	0.0012	0.0090
59	0.0005	0.0013	0.0094
60	0.0006	0.0014	0.0097
61	0.0007	0.0015	0.0101
62	0.0007	0.0016	0.0105
63	0.0009	0.0018	0.0108
64	0.0010	0.0020	0.0111
65	0.0012	0.0021	0.0115
66	0.0013	0.0023	0.0118
67	0.0015	0.0025	0.0122
68	0.0018	0.0027	0.0127
69	0.0021	0.0030	0.0133
70	0.0024	0.0032	0.0139
71	0.0027	0.0035	0.0145
72	0.0030	0.0038	0.0151
73	0.0037	0.0042	0.0160
74	0.0044	0.0046	0.0168
75	0.0050	0.0050	0.0177
76	0.0057	0.0054	0.0186
77	0.0064	0.0059	0.0195
78	0.0077	0.0064	0.0211
79	0.0089	0.0070	0.0226
80	0.0102	0.0076	0.0242
81	0.0115	0.0083	0.0258
82	0.0128	0.0090	0.0273
83	0.0147	0.0098	0.0294
84	0.0167	0.0107	0.0315
85	0.0186	0.0117	0.0336
86	0.0206	0.0127	0.0358
87	0.0225	0.0139	0.0379
88	0.0239	0.0152	0.0394
89	0.0252	0.0165	0.0410
90	0.0265	0.0180	0.0426
91	0.0278	0.0197	0.0442
92	0.0292	0.0215	0.0458
93	0.0305	0.0235	0.0474
94	0.0318	0.0257	0.0490
95	0.0331	0.0280	0.0506
96	0.0344	0.0307	0.0521
97	0.0358	0.0336	0.0537
98	0.0371	0.0367	0.0553
99	0.0384	0.0403	0.0569
100	0.0397	0.0441	0.0585

Leslie et al. 2011 [50] for hip fracture, unpublished data from the Canadian Multicentre Osteoporosis Study for vertebral fractures [51] (consistent with a previously published cost-effectiveness analysis of denosumab in a Canadian setting [52]), and Leslie et al. 2009 [53] for other fracture (which included non-hip, non-vertebral fractures)

**Table 6:** Fitted regression parameters (selected parameters with the best fit according to AIC shown in bold)

Parametric model (parameters)	Parameter 1 (SE)	Parameter (SE)	Parameter 3 (SE)	AIC	BIC
**Hip fracture-romosozumab/alendronate**					
** Exponential (rate)**	** − 10.770 (0.156)**	**-**	**-**	**967.1513**	972.775
Weibull (shape/scale)	** − **0.095 (0.149)	11.149 (0.647)	-	968.732	979.9793
Gompertz (shape/rate)	0.000 (0.000)	** − **10.680 (0.273)	-	969.0073	980.2546
Lognormal (meanlog/sdlog)	13.067 (0.877)	1.109 (0.135)	-	969.4386	980.6859
Log-logistic (shape/scale)	** − **0.089 (0.148)	11.112 (0.642)	-	968.7473	979.9946
Gamma (shape/rate)	** − **0.097 (0.151)	** − **11.204 (0.724)	-	968.7274	979.9747
Generalized gamma (mu/sigma/Q)	10.910 (2.602)	** − **0.381 (25.767)	1.618 (41.705)	970.7182	987.5891
**Hip fracture-alendronate**					
Exponential (rate)	** − **10.286 (0.123)	-	-	1491.744	1497.369
Weibull (shape/scale)	0.157 (0.115)	9.802 (0.343)	-	1491.991	1503.239
Gompertz (shape/rate)	0.000 (0.000)	** − **10.371 (0.222)	-	1493.548	1504.796
** Lognormal (meanlog/sdlog)**	**10.802 (0.440)**	**0.757 (0.105)**	**-**	**1490.05**	1501.298
Log-logistic (shape/scale)	0.168 (0.115)	9.753 (0.339)	-	1491.83	1503.078
Gamma (shape/rate)	0.170 (0.123)	** − **9.674 (0.426)	-	1491.899	1503.147
Generalized gamma (mu/sigma/Q)	11.190 (0.655)	1.124 (0.651)	** − **0.633 (1.403)	1491.756	1508.628
**Nonvertebral fracture-romosozumab/alendronate**					
** Exponential (rate)**	** − 9.261 (0.075)**	**-**	**-**	**3654.774**	3660.398
Weibull (shape/scale)	** − **0.095 (0.071)	9.491 (0.199)	-	3654.925	3666.172
Gompertz (shape/rate)	0.000 (0.000)	** − **9.199 (0.131)	-	3656.48	3667.728
Lognormal (meanlog/sdlog)	10.224 (0.246)	0.911 (0.062)	-	3660.579	3671.826
Log-logistic (shape/scale)	** − **0.070 (0.070)	9.377 (0.194)	-	3655.423	3666.67
Gamma (shape/rate)	** − **0.103 (0.075)	** − **9.572 (0.250)	-	3654.851	3666.098
Generalized gamma (mu/sigma/Q)	9.333 (0.344)	** − **0.589 (2.922)	2.030 (5.932)	3656.506	3673.377
**Nonvertebral fracture-alendronate**					
Exponential (rate)	** − **9.048 (0.068)	-	-	4363.016	4368.64
Weibull (shape/scale)	** − **0.096 (0.064)	9.263 (0.168)	-	4362.684	4373.933
** Gompertz (shape/rate)**	** − 0.00034 (0.00018)**	** − 8.871 (0.116)**	**-**	**4361.946**	4373.195
Lognormal (meanlog/sdlog)	9.798 (0.202)	0.864 (0.056)	-	4365.103	4376.351
Log-logistic (shape/scale)	** − **0.063 (0.063)	9.119 (0.162)	-	4362.252	4373.501
Gamma (shape/rate)	** − **0.102 (0.069)	** − **9.337 (0.216)	-	4362.791	4374.039
Generalised gamma (mu/sigma/Q)	9.474 (0.291)	0.424 (0.314)	0.608 (0.374)	4364.046	4380.919

*SE*, standard error; *AIC*, Akaike information criterion; *BIC*, Bayesian information criterion

**Table 7:** Relative risks of mortality following fracture

Age (years)	Hip fracture 1st year	Hip fracture 2nd and following years	Vertebral fracture 1st year	Vertebral fracture 2nd and following years	Other fracture 1st year
50–59	21.3	3.4	3.4	2.1	2.8
60–69	5.1	8.3	2.5	1.7	2.2
70–79	3.8	3.9	1.7	1.6	1.7
80–89	2.0	1.4	1.0	1.0	1.7
90 +	1.5	1.4	0.8	0.8	1.4

Source: Morin et al. 2011 [2]

**Table 8:** Societal costs

Parameter	Value	Source
**Hourly wages**		
Hourly wage for females aged 55 years and older	$25.95	[54]
Hourly wage for females and males aged 15 years and older	$26.92	
**Hours worked per year**		
Hours worked in full-time employment	1920	[54]
Hours worked in part-time employment	960	
**Workforce participation rates**		
**Proportion of people working full time**		
50–54 years	64.5%	[55]
55–59 years	53.0%	
60–64 years	33.4%	
65–69 years	11.0%	
70 + years	2.1%	
**Proportion of people working part time**		
50–54 years	14.1%	
55–59 years	15.2%	
60–64 years	14.7%	
65–69 years	9.9%	
70 + years	3.1%	
**Time off work following fracture (years)**		
Hip fracture	1.000	Clinical experts
Vertebral fracture	0.250	
Other fracture	0.115	
**Hours of informal care per week following hip fracture**		
2-month post-fracture	5.2	[56]
3–12-month post-fracture	4.3	
**Calculated indirect costs per fracture**		
**Hip fracture**		
50–54 years	$41,890	[54]
55–59 years	$36,458	
60–64 years	$26,578	
65–69 years	$14,188	
70 + years	$8059	
**Vertebral fracture**		
50–54 years	$8910	
55–59 years	$7552	
60–64 years	$5082	
65–69 years	$1984	
70 + years	$452	
**Other fracture**		
50–54 years	$4098	
55–59 years	$3474	
60–64 years	$2337	
65–69 years	$913	
70 + years	$208	

## References

[1] Cooper C (1997). The crippling consequences of fractures and their impact on quality of life. Am J Med..

[2] Morin S, Lix LM, Azimaee M, Metge C, Caetano P, Leslie WD (2011). Mortality rates after incident non-traumatic fractures in older men and women. Osteoporos Int..

[3] Brown JP, Adachi JD, Schemitsch E (2021). Mortality in older adults following a fragility fracture: real-world retrospective matched-cohort study in Ontario. BMC Musculoskelet Disord.

[4] Hopkins RB, Burke N, Von Keyserlingk C (2016). The current economic burden of illness of osteoporosis in Canada. Osteoporos Int..

[5] Kendler DL, Adachi JD, Brown JP (2021). A scorecard for osteoporosis in Canada and seven Canadian provinces. Osteoporos Int..

[6] van Geel TA, van Helden S, Geusens PP, Winkens B, Dinant GJ (2009). Clinical subsequent fractures cluster in time after first fractures. Ann Rheum Dis.

[7] Camacho PM, Petak SM, Binkley N (2020). American Association of Clinical Endocrinologists/American College of Endocrinology Clinical Practice Guidelines for the diagnosis and treatment of postmenopausal osteoporosis-2020 update. Endocr Pract..

[8] Eastell R, Rosen CJ, Black DM, Cheung AM, Murad MH, Shoback D (2019). Pharmacological management of osteoporosis in postmenopausal women: an Endocrine Society clinical practice guideline. J Clin Endocrinol Metab.

[9] Kanis JA, Harvey NC, McCloskey E (2020). Algorithm for the management of patients at low, high and very high risk of osteoporotic fractures. Osteoporos Int..

[10] Adachi JD, Brown JP, Schemitsch E (2021). Fragility fracture identifies patients at imminent risk for subsequent fracture: real-world retrospective database study in Ontario, Canada. BMC Musculoskelet Disord.

[11] PHAC (2020) Osteoporosis and related fractures in Canada - report from the Canadian chronic disease surveillance system. Public Health Agency of Canada. 1–83. 978–0–660–33153–9

[12] Papaioannou A, Morin S, Cheung AM (2010). 2010 clinical practice guidelines for the diagnosis and management of osteoporosis in Canada: summary. CMAJ.

[13] Hayes KN, Ban JK, Athanasiadis G, Burden AM, Cadarette SM (2019). Time trends in oral bisphosphonate initiation in Ontario, Canada over 20 years reflect drug policy and healthcare delivery changes. Osteoporos Int..

[14] Durden E, Pinto L, Lopez-Gonzalez L, Juneau P, Barron R (2017). Two-year persistence and compliance with osteoporosis therapies among postmenopausal women in a commercially insured population in the United States. Arch Osteoporos.

[15] Kim M, Park A, McGrath L, Wiener C, Balasubramanian A, McDermott M (2020). Trends in osteoporosis treatment uptake and persistence among postmenopausal women in the U.S., 2010–2015. Presented at ENDO 2020 Online. J Endocr Soc.

[16] Koller G, Goetz V, Vandermeer B, Homik J, McAlister FA, Kendler D (2020). Persistence and adherence to parenteral osteoporosis therapies: a systematic review. Osteoporos Int.

[17] Liu J, Guo H, Rai P, Pinto L, Barron R (2018). Medication persistence and risk of fracture among female Medicare beneficiaries diagnosed with osteoporosis. Osteoporos Int.

[18] Shoback D, Rosen CJ, Black DM, Cheung AM, Murad MH, Eastell R (2020). Pharmacological management of osteoporosis in postmenopausal women: an endocrine society guideline update. J Clin Endocrinol Metab.

[19] Saag KG, Petersen J, Brandi ML (2017). Romosozumab or alendronate for fracture prevention in women with osteoporosis. N Engl J Med..

[20] Hagino H, Tanaka K, Silverman S (2021). Cost-effectiveness of romosozumab versus teriparatide for severe postmenopausal osteoporosis in Japan. Osteoporos Int.

[21] Soreskog E, Lindberg I, Kanis JA (2021). Cost-effectiveness of romosozumab for the treatment of postmenopausal women with severe osteoporosis at high risk of fracture in Sweden. Osteoporos Int.

[22] Black DM, Arden NK, Palermo L, Pearson J, Cummings SR (1999). Prevalent vertebral deformities predict hip fractures and new vertebral deformities but not wrist fractures. Study of osteoporotic fractures research group. J Bone Miner Res..

[23] Canadian Agency for Drugs and Technologies in Health (CADTH). Guidelines for the economic evaluation of health technologies: Canada — 4th Edition 2017. [cited 7th October, 2020]. Available from: https://www.cadth.ca/dv/guidelines-economic-evaluation-health-technologies-canada-4th-edition

[24] Amgen Canada Inc. EVENITY® romosozumab injection Product Monograph. Mississauga: Amgen Canada Inc.; 17 Jun 2019

[25] Qaseem A, Forciea MA, McLean RM, Denberg TD (2017). Treatment of low bone density or osteoporosis to prevent fractures in men and women: a clinical practice guideline update from the American College of Physicians. Ann Intern Med.

[26] Jönsson B, Christiansen C, Johnell O, Hedbrandt J (1995). Cost-effectiveness of fracture prevention in established osteoporosis. Osteoporos Int.

[27] Svedbom A, Hadji P, Hernlund E (2019). Cost-effectiveness of pharmacological fracture prevention for osteoporosis as prescribed in clinical practice in France, Germany, Italy, Spain, and the United Kingdom. Osteoporos Int.

[28] Zethraeus N, Borgstrom F, Strom O, Kanis JA, Jonsson B (2007). Cost-effectiveness of the treatment and prevention of osteoporosis-a review of the literature and a reference model. Osteoporos Int..

[29] Jonsson B, Strom O, Eisman JA (2011). Cost-effectiveness of denosumab for the treatment of postmenopausal osteoporosis. Osteoporos Int..

[30] O’Hanlon CE, Parthan A, Kruse M (2017). A model for assessing the clinical and economic benefits of bone-forming agents for reducing fractures in postmenopausal women at high, near-term risk of osteoporotic fracture. Clin Ther.

[31] Parthan A, Kruse M, Yurgin N, Huang J, Viswanathan HN, Taylor D (2013). Cost-effectiveness of denosumab versus oral bisphosphonates for postmenopausal osteoporosis in the US. Appl Health Econ Health Policy..

[32] Parthan A, Kruse M, Agodoa I, Silverman S, Orwoll E (2014). Denosumab: a cost-effective alternative for older men with osteoporosis from a Swedish payer perspective. Bone..

[33] Hiligsmann M, Kanis JA, Compston J (2013). Health technology assessment in osteoporosis. Calcif Tissue Int..

[34] Barrionuevo P, Kapoor E, Asi N (2019). Efficacy of pharmacological therapies for the prevention of fractures in postmenopausal women: a network meta-analysis. J Clin Endocrinol Metab.

[35] Amgen. ARCH Clinical Study Report (data on file)

[36] Black DM, Schwartz AV, Ensrud KE (2006). Effects of continuing or stopping alendronate after 5 years of treatment: the Fracture Intervention Trial Long-term Extension (FLEX): a randomized trial. JAMA.

[37] Statistics Canada. Life tables, Canada, provinces and territories 2016 to 2018. (Complete life tables, female, Canada) 2020. [cited November 11th, 2020]. Available from: https://www150.statcan.gc.ca/n1/pub/84-537-x/2019002/xls/2016-2018_Tbl-eng.xlsx.

[38] Svedbom A, Borgstöm F, Hernlund E (2018). Quality of life for up to 18 months after low-energy hip, vertebral, and distal forearm fractures—results from the ICUROS. Osteoporos Int.

[39] Tarride J-E, Burke N, Leslie WD (2016). Loss of health related quality of life following low-trauma fractures in the elderly. BMC Geriatr.

[40] Guertin JR, Feeny D, Tarride JE (2018). Age- and sex-specific Canadian utility norms, based on the 2013–2014 Canadian community health survey. CMAJ.

[41] Ontario Drug Benefit Formulary [Internet]. 2020 [cited November 11th 2020]. Available from: https://www.formulary.health.gov.on.ca/formulary/

[42] Ministry of Health and Long Term Care. Schedule of benefits: physician services under the health insurance act. Government of Ontario. 2020. Queens Printer for Ontario

[43] Metge CAM, Lix LM, Morin S, Caetano P, Leslie WD (2010) Using cost-of-illness analysis to describe the direct cost burden of fracture: estimates of potential savings from prevention. Can J Clin Pharmacol 17(1):e121 (abstract with supplementary material);17[2]

[44] Goeree R, Blackhouse G, Adachi J (2006). Cost-effectiveness of alternative treatments for women with osteoporosis in Canada. Curr Med Res Opin.

[45] AdvantAge Ontario. About long term care homes 2020. [cited November 11th, 2020]. Available from: http://www.advantageontario.ca/AAO/Content/Resources/Consumers/About_Long_Term_Care.aspx?WebsiteKey=00bad89a-d342-4c6e-bc0c-b4b493c5462c

[46] inflation.eu. Historic inflation Canada - CPI inflation 2020. [cited September 15th, 2020]. Available from: https://www.inflation.eu/en/inflation-rates/canada/historic-inflation/cpi-inflation-canada.aspx

[47] Fatoye F, Smith P, Gebrye T, Yeowell G (2019). Real-world persistence and adherence with oral bisphosphonates for osteoporosis: a systematic review. BMJ open..

[48] Ontario Nurses’ Association. Nursing homes template contract 2019–2021. [cited November 11th, 2020]. Available from: https://www.ona.org/wp-content/uploads/ona_nursinghomestemplatecontract_20210630.pdf

[49] Briggs A, Claxton K, Sculpher M (2006) Decision modelling for health economic evaluation. Oxford University Press, New York

[50] Leslie WD, Lix LM, Langsetmo L (2011). Construction of a FRAX® model for the assessment of fracture probability in Canada and implications for treatment. Osteoporos Int.

[51] Canadian Multi-Centre Osteoporosis Study (CaMos) (2009) Unpublished analysis

[52] Chau D, Becker D, Coombes M, Ioannidis G, Adachi J, Goeree R (2012). Cost-effectiveness of denosumab in the treatment of postmenopausal osteoporosis in Canada. J Med Econ.

[53] Leslie WD, Morin S, Azimaee M, Lix LM, Metge C, Caetano P (2009) Secular decreases in osteoporotic fracture rates 1986–2006- a population-based analysis. 2009–10, ISPOR Europe; Paris, France. Value Health 12:7

[54] Statistics Canada. Employee wages by occupation, annual 2020. [cited November 11^th^, 2020]. Available from: https://www150.statcan.gc.ca/t1/tbl1/en/tv.action?pid=1410030701

[55] Statistics Canada. Labour force characteristics by sex and detailed age group 2020. [cited September 3^rd^, 2020]. Available from: https://www150.statcan.gc.ca/t1/tbl1/en/tv.action?pid=1410001801&pickMembers%5B0%5D=1.6&pickMembers%5B1%5D=2.1

[56] Wiktorowicz M, Goeree R, Papaioannou A, Adachi JD, Papadimitropoulos E (2001). Economic implications of hip fracture: health service use, institutional care and cost in Canada. Osteoporos Int.

